# Supplementary material for: Efficacy of the CDK7 Inhibitor on EMT-Associated Resistance to 3rd Generation EGFR-TKIs in Non-Small Cell Lung Cancer Cell Lines
Source: Cells. 2020 Dec 3;9(12):2596. doi: 10.3390/cells9122596 (PMC7761809; doi:10.3390/cells9122596)
Supplement: Supplementary file 1 [file cells-09-02596-s001.pdf]

Efficacy of the CDK7 inhibitor on EMT-associated resistance to 3rd generation EGFR-TKIs in non-small cell lung cancer cell lines

Wonjun Ji[1], Yun Jung Choi[2], Myoung-Hee Kang[2], Ki Jung Sung[2], Dong Ha Kim[2], Sangyong Jung[3], Chang-Min Choi[1,4], Jae Cheol Lee[4,*], Jin Kyung Rho[5,*]

[1]Department of Pulmonology and Critical Care Medicine, Asan Medical Center, University of Ulsan, College of Medicine, Seoul 05505, South Korea.

[2]Asan Institute for Life Sciences, Asan Medical Center, University of Ulsan, College of Medicine, Seoul 05505, South Korea.

[3]Department of Biomedical Sciences, Asan Medical Center, AMIST, University of Ulsan, College of Medicine, Seoul 05505, South Korea.

[4]Department of Oncology, Asan Medical Center, University of Ulsan, College of Medicine, Seoul 05505, South Korea.

[5]Department of Convergence Medicine, Asan Medical Center, University of Ulsan, College of Medicine, Seoul 05505, South Korea.

*Co-corresponding authors:

Jae Cheol Lee

Department of Oncology, University of Ulsan College of Medicine, 88, Olympic-ro 43-gil, Songpa-gu, Seoul 05505, South Korea. Tel: +82 2 3010 3208; Fax: +82 2 3010 6961; E-mail: jclee@amc.seoul.kr

Jin Kyung Rho

Department of Convergence Medicine, University of Ulsan College of Medicine, 88, Olympic-ro 43-gil, Songpa-gu, Seoul 05505, South Korea. Tel: +82 2 3010 2974; Fax: +82 2 3010 6961;

E-mail: jkrho@amc.seoul.kr

Materials and Methods

RNA sequencing and Kyoto Encyclopedia of Genes and Genomes (KEGG) pathway mapping analysis

Each cell was pelleted, and total RNA was extracted using an RNeasy Mini Kit (Qiagen, Hilden, Germany). Genomic DNA was eliminated, and the RNA integrity was verified using the Agilent 2100 Bioanalyzer (Agilent Technologies, Santa Clara, CA, USA). RNA Sequencing was performed by a specialized company (Macrogen, Seoul, Korea) using the TruSeq RNA Sample Prep Kit v2 and HiSeq. 2500 platform (Illumina, San Diego, CA, USA). The sequenced reads were trimmed using TrimMomatic 0.32. The trimmed reads were mapped to the hg19 reference genomes using HISAT (version 2.0.5) and Bowtie2. Expression levels were measured as kilobase of transcript per million mapped reads using StringTie (version 1.3.3b). The potential targets of the differentially expressed genes were analyzed by the KEGG program. In the KEGG pathway enrichment analysis, enriched pathways were identified according to $p < 0.05$.

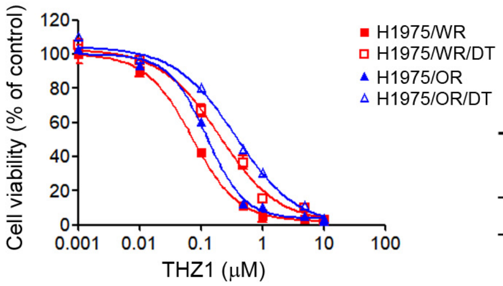

Drug	IC_{50} values (nM, mean ± S.D.)			
	H1975/WR	H1975/WRDT	H1975/OR	H1975/OR/DT
THZ1	72.4 (±18.8)	200.9 (±31.6)	124.3 (±17.9)	368.9 (±30.0)

Supplementary Figure S1. IC_{50} values of THZ1-tolerant cells. Cells were treated with the indicated doses of THZ1 for 72 h, and cell viability was determined by an MTT assay. IC50 values were calculated with GraphPad software.

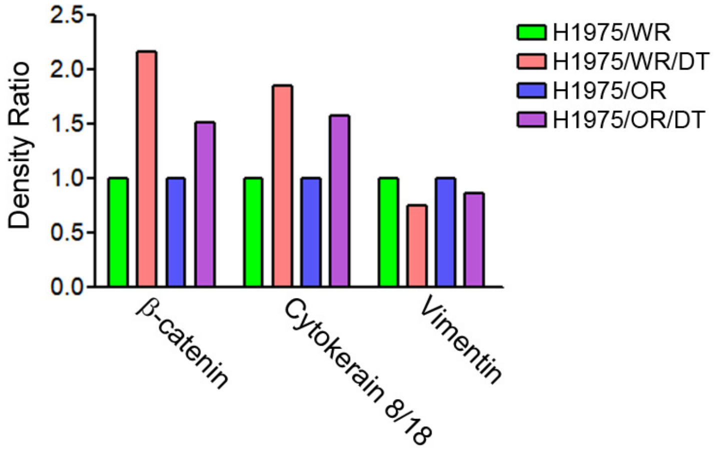

Supplementary Figure S2. Changes of EMT-related molecules in THZ1-tolerant cells. Densitometric analysis was performed for the data shown in Figure 5B with ImageJ software from NIH (http://rsb.info.nih.gov/nih-image/).

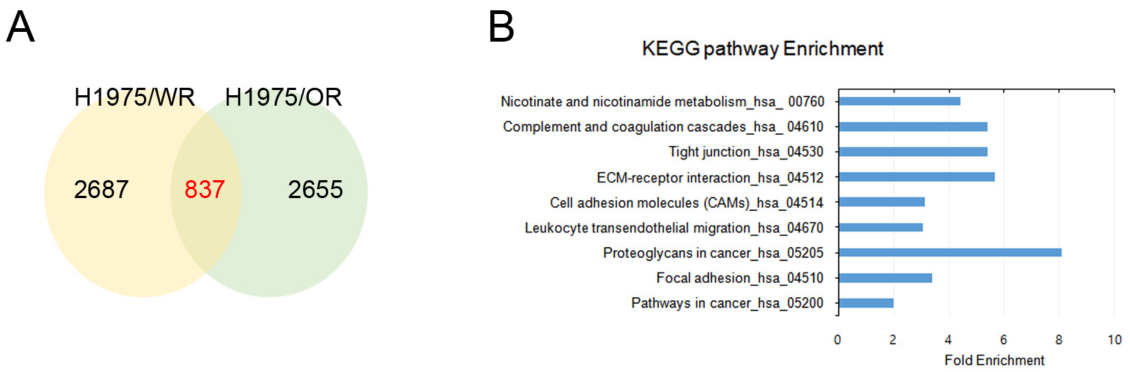

Supplementary Figure S3. The analysis of RNA-seq data from parental and 3rd-generation EGFR-TKIs-resistant cells. (**A**) The Venn diagram showing over two-fold up- and down-regulation of mRNAs in resistant cells. (**B**) KEGG analysis of the enrichment of differentially expressed genes between parental (H1975) and resistant cells (H1975/WR and H1975/OR) in specific pathways.

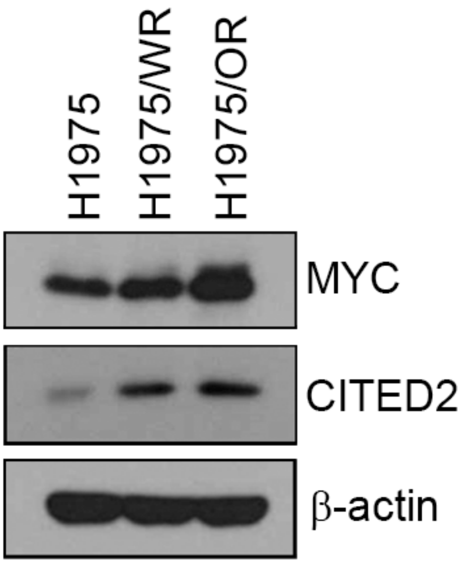

Supplementary Figure S4. Comparisons between the basal levels of parental and both resistant cell lines. MYC and CITED2 expression were detected by western blotting.
